# Time to recovery from obstetric fistula and determinants in Gondar university teaching and referral hospital, northwest Ethiopia

**DOI:** 10.1186/s12905-018-0700-3

**Published:** 2019-01-07

**Authors:** Leltework Yismaw, Kassahun Alemu, Abebaw Addis, Muluneh Alene

**Affiliations:** 1 Department of Public Health, Debre Markos University, Debre Markos, Ethiopia; 2 Department of Epidemiology and Biostatistics, Institute of Public Health, University of Gondar, Gondar, Ethiopia; 3 Department of Reproductive Health, Institute of Public Health, University of Gondar, Gondar, Ethiopia; 4 Department of Statistics, Mizan-Tepi University, Teppi, Ethiopia

**Keywords:** Obstetric fistula, Recovery time, Determinants, Ethiopia

## Abstract

**Background:**

Obstetric fistula is an abnormal connection between the vagina and rectum and/or bladder, which leads to continuous urinary or fecal incontinence. It is a serious problem in the world poorest countries, where most mothers give birth without any medical care. In most cases obstetric fistula is preventable and can be treated successfully, if it is carried out by a competent surgeon with a good follow-up of postoperative care. However, there remains to explore more on the duration of obstetric fistula recovery and determinant factors. The aim of this study was to estimate the average recovery time of obstetric fistula and to identify its determinants in Gondar University teaching and referral hospital, northwest Ethiopia.

**Method:**

A retrospective follow up study was conducted at Gondar University teaching and referral hospital. A total of 612 fistula cases were included in the study and simple random sampling technique was applied to select the study subjects. Kaplan-Meier and log rank test were computed to explore the data. Weibull regression survival model with univariate frailty was done to identify the determinant factors of time to recovery.

**Results:**

Of 612 fistula patients, 539(88.07%) were recovered. The Average (median) recovery time was 5.14 (IQR = 3.14, 9.14) weeks. Using Antibiotic (AHR = 1.49, 95% CI = 1.11–2.01), having history of antenatal care (ANC) (AHR = 1.95, 95% CI = 1.39–2.73), being literate (AHR = 2.23, 95% CI = 1.62–3.06), duration of bladder catheterization (AHR = 0.93, CI = 0.90–0.95) and being multiparous (AHR = 1.51, 95% CI = 1.17–1.96) were a significant predictors of the rate of recovery. Also, underweight (AHR = 0.45, 95% CI = 0.30–0.68), overweight (AHR = 0.56, 95% CI = 0.41–0.76), being obese (AHR = 0.41, 95% CI = 0.21–0.80), having extensive fistula (AHR = 0.82, 95% CI = 0.73–0.91), large fistula (AHR = 0.42, 95% CI = 0.23–0.78), medium width (AHR = 0.62, 95% CI = 0.43–0.91) and large width (AHR = 0.42, 95% CI = 0.23–0.78) were statistically significant predictors of the rate of recovery from fistula patients.

**Conclusion:**

The average recovery time from obstetric fistula patients was 5.14 weeks. Small Length and width of fistula, patients’ educational status (literacy), antibiotic use, history of antenatal care visits, normal BMI, short period catheterization and being multiparous were the significant determinate variables which shorten the recovery time of obstetric fistula.

## Background

Obstetric fistula is abnormal connection between the rectum and vagina (recto vaginal fistula) or between the bladder and vagina (vesico vaginal fistula). Obstructed and prolonged labors are the most common causes of obstetric fistula. It is a serious problem in the world’s poorest countries, where most mothers give birth without any medical care [1–3]. More than 2 million women’s live with obstetric fistula in the developing country, and 50,000 to 100,000 new cases develop in each year [4, 5]. Sub-Saharan Africa and south Asia are the regions where the highest obstetric fistula patients exit, which are estimated to be more than 1 million. There are over 6000 new cases per year in these two world regions. The prevalence of obstetric fistula is 0.29 per 1000 women of reproductive age in the developing world. The prevalence of obstetric fistula in sub-Saharan Africa and south Asia was 1.6 and 1.2 per 1000 women of reproductive age respectively [6].

In Ethiopia, there were nearly 142,387 obstetric fistula patients in 2013. In this setting 10.6 per 1000 women who ever given birth experiences obstetric fistula in their life [7]. Amhara region is the area where the highest prevalence of obstetric fistula exists (7 per 1000 women) next to Tigray region [8]. Obstetric fistula has a devastating impact on women’s physical, social and psychological health [9]. In most cases obstetric fistula is preventable and can be treated successfully, if it is carried out by a competent surgeon with a good follow-up of postoperative care [10].

The recovery time of obstetric fistula patients depends on different factors. From the total patients’ who got surgical treatment 81.7% were physically cured on the average time of 4.64 weeks [11]. The success rates for short-term fistula were 89% with 2–4 weeks post-surgery [12].

The size and scarring of fistula are significant factors for incontinence and failure of closure. Women’s who have large size of fistula are less likely to be continence compared to those who have small size of fistula [11–15]. Women with antenatal care follow-up, delivery at health institution, labor less than 2 days, vaginal delivery, height ≥ 150 cm and weight less than 50 kg had shorter recovery time than their counter group [11]. Patients who got treatment within 3 months after developing fistula had better closure outcome than patients who got treatment more than 3 months [16]. Patients who took antibiotic prior to surgery had better outcome than patients who didn’t take antibiotics. Patients catheterized for 12 up to 14 days had significantly greater likelihood of residual incontinence than those catheterized for 10 days [15].

The previous studies have mainly emphasized on the outcome of fistula surgery without considering the recovery time. However, there remains to explore more on the duration of recovery from obstetric fistula and its determinant factors after attaining the treatment. Thus, this study was aimed to estimate the average recovery time of obstetric fistula and its determinant factors. The study is important to bring information’s for physician, which is helpful to cure patients within a short period of time and in turn it enables the women to return from the previous normal life and to have rest.

## Methods

### Study design and setting

An institutional based retrospective follow-up study was conducted to estimate the time to recovery from obstetric fistula and its determinant factors between January, 2010 to march, 2017 in Gondar University teaching and referral hospital. Data were extracted from hospital registry of patient chart on obstetric fistula from initial date of entry until complete the follow-up.

Gondar town is situated in north Gondar zone, which is the most populated zone (population of 3,061,220) in Amhara region. Gondar University teaching and referral hospital is found in Gondar town [10]. Women And Health Alliance International (WAHA International) partnered with Gondar University teaching and referral hospital provide fistula repair surgery and integrate fistula care in 2010. WAHA International in collaboration with fistula foundation and United Nation Population Fund (UNFPA) completed 70 bed ward facility which includes two operating theatres and physiotherapy unit which aims to treat around 100 patients per month [17].

### Study population and sample size

Patients with fistula who got the chance to be treated in Gondar University teaching and referral hospital were the study population. All patients who got treatment in Gondar University teaching and referral hospital from January 2010 to March 2017 were included in the study. Transferred out patients and patients who had incomplete chart were excluded from the study.

The sample size was determined by using sample size determination formula for time to event data [18]. The following assumptions are considered for the sample size determination: *Z*_*α*/2_ is the critical value of standard normal distributed variable at 5 % significance level, *Z*_*β*_ is the critical value of standard normal distributed variable at 20 % of *β* and *β* is the probability of type two error, b is log (hazard ratio), *p*_1_ is the proportion of number of patients in the first category, *p*_2_ is the proportion of number of patients in the second category and d represents proportion of the event. The proportion of recovered patients from obstetric fistula (d = 0.817) was taken from a previous study conducted at Yirgalem Hamlin fistula hospital [11]. Finally the required sample was 612 patients. Two thousand twenty seven fistula patients were treated in the hospital, from January 2010 to march 2017, of these 612 patients were selected randomly.

### Outcome and predictor variables

The outcome variable of this study was time to recovery from obstetric fistula. Socio demographic variables (residence, marital status, age, body mass index (BMI), educational status and patients economically dependence) were used as predictor variables. Similarly obstetrics factors such as antenatal care follow-up, parity, delivery place, mode of delivery and duration of labor were considered to predict the outcome variable. Furthermore fistula characteristics like duration between the onset of the fistula and time of surgical treatment, width of fistula hole and Length of fistula hole), pre and post-operative care factor (antibiotic use and duration of bladder catheterization) were the variables used to estimate the time to recovery from obstetric fistula. Recovery is defined as patients able to control the urine and the feces as judged by the physician. Time to recovery was defined as time from enrollment to recover, whereas patients who didn’t recover during the study period, lost at follow up or death were considered as censored observations.

### Statistical analysis

The collected data was coded and entered to EpiData version 3.1 and exported to STATA version 14 for analysis. Survival analysis was employed to estimate the average (median) recovery time and to identify determinants factors. Survival analysis is a collection of statistical procedures for data analysis when the outcome variable of interest is time until an event occurs. The data was explored using Kaplan-Meier and log rank test. Kaplan-Meier test was needed to estimate the distribution of recovery time and to observe the experience of recovery time among different levels of categorical variables. The Log rank test is a non-parametric method that compares two or more groups in regards of survival experience. Under the null hypothesis, long rank test assumes that the survival probability is equal at each levels of categorical variables which means that no association between the event and the group.

In this study parametric method of survival analysis was employed. Exponential, Weibull and Gompertz distributions were considered to select the appropriate model. The procedure of model building was as follows: First, separate models were fitted for each distribution. Second, the first step was repeated after incorporating univariate frailty. Finally the parsimonious model was selected after carried out different model comparison criterion. The advantage of using a parametric distribution is that one can reliably predict the time to event, after the period during which events occurred for the observed data. In the data the total variability of the hazard function mainly resulted from two sources. The first is the variability that comes from observable factors which is already included in the model and the other is from unobservable variables or variables which are not included the model. In the analysis unobserved covariates should be considered, unless it produces biased estimates. Overcoming the problem of heterogeneity by incorporating both observable and non-observable factors is called frailty in survival analysis [19].

## Results

**Fig. 1:**
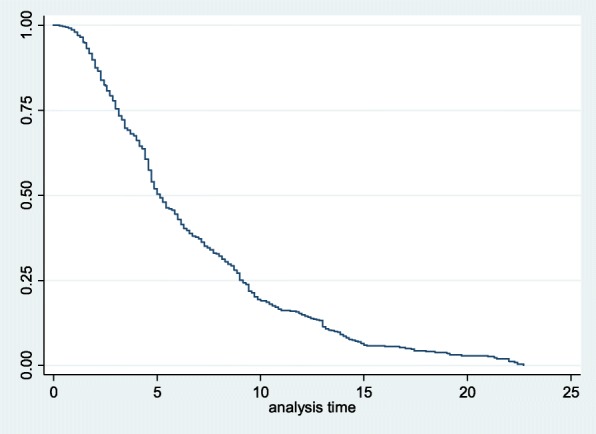
Kaplan-Meier estimator curve for the recovery time of obstetric fistula patients at Gondar University teaching and referral hospital (January, 2010–March, 2017)

This study included 612 obstetric fistula patients, which were attained from Gondar University teaching and referral hospital. Among the included patients 88.07% (95% CI = 85.25–90.42) were recovered from obstetric fistula with a median time of 5.14 (IQR = 3.14, 9.14) weeks (Fig. 1). The Log-rank test shows a higher recovery time for rural patients than for urban patients. Moreover the long rank test shows that the recovery time was significantly different according to the patient’s body mass index (Table 1).

**Table 1:** Comparisons of time to recovery among different levels of predictor variables using log-rank test from obstetric fistula patients at Gondar university teaching and referral hospital (January, 2010–March, 2017)

Variables	Frequency (%)	Median recovery time in week	Log rank test	
			X^2^	*P*-value
Residence				
Urban	122 (19.9%)	4	28.00	< 0.001
Rural	490 (80.1%)	5.9		
Marital status				
Single	27 (4.4%)	5.1	3. 7	0.452
Married	387 (63.3%)	4.9		
Divorced	114 (18.7%)	5.4		
Widowed	32 (5.2%)	5.9		
Separate	51 (8.4%)	7.3		
BMI				
Normal weight	212 (34.9%)	3.6	76.1	< 0.001
Under weight	98 (16.1%)	8.9		
Over weight	273 (44.9%)	5.3		
Obesity	28 (4.6%)	10.4		
Educational status				
Illiterate	394 (64.4%)	7.3	174.2	< 0.001
Literate	218 (35.6%)	3.4		
Economical dependence				
Dependent	290 (47.6%)	7.3	49.7	< 0.001
Independent	319 (52.4%)	4.4		
Parity				
Primiparous	299 (49.0%)	5.9	8.6	0.003
Multiparous	311 (51.0%)	4.9		
Antenatal care				
Yes	303 (49.6%)	3.6	166.7	< 0.001
No	308 (50.4%)	8.4		
Delivery place				
Home	293 (48.0%)	6.6	22.2	< 0.001
Health service	317 (52.0%)	4.4		
Mode of delivery				
Vaginal	454 (74.4%)	5.3	0.8	0.359
Non-vaginal	156 (25.6%)	4.7		
Outcome of delivery				
Still-birth	256 (41.8%)	6.4	10.6	< 0.001
Alive	347 (57.6%)	4.6		
Antibiotic use				
Use	357 (61.5%)	4.4	90.4	< 0.001
Don’t use	224 (38.6%)	8.6		
Physiotherapy				
No	363 (59.5%)	6.6	45.7	< 0.001
Yes	247 (40.5%)	4		
Length of fistula				
Small (< 2 cm.)	332 (54.3%)	4	117.2	< 0.001
Medium (2–3.9 cm.)	99 (16.2%)	5.3		
Large (4–5.9 cm.)	131 (21.4%)	8.4		
Extensive (>/=6 cm.)	50 (8.2%)	10.9		
Width of fistula				
Small (< 2 cm.)	330 (53.9%)	4	148.0	< 0.001
Medium (2–3.9 cm.)	133 (21.7%)	6.7		
Large (4–5.9 cm.)	118 (19.3%)	8.9		
Extensive (>/=6 cm.)	31 (5.1%)	9.7		

The long rank test shows a significant difference between illiterate and literate patients in recovery time. Patients who didn’t have formal education took longer time to recover as compared to with those which have formal education. Also there is a statistical significant difference in recovery time between economical dependent and independent patients. Economically dependent patients took longer recovery time than economically independent patients (Table 1). The long rank test shows there is a significance deference between primiparous and multiparous women’s in recovery time. A women giving birth for the first time were taken longer recovery time than women’s who give birth two times and above. Also the recovery time of patients who had antenatal care visit was shorter than patients who had not antenatal care visit (Table 1).

Patients who were delivered at home had longer recovery time than those who delivered at health institution. Women who give alive birth had short recovery time compare with patients who had given still birth. Antibiotic treatment helps the patient to have shorter recovery time compared to patients who didn’t use antibiotic. Also taking physiotherapy as supportive treatment helped to reduce recovery time. Furthermore the log rank test result shows, there is a statistical significance difference among each category of both length and width of fistula. As the length and width of fistula increases, the average (median) recovery time also increases (Table 1).

Multiple covariate analysis showed that antenatal care visit, BMI, parity, antibiotic use, duration of bladder catheterization, length of fistula hole, width of fistula hole and educational status were significantly associated with recovery time of obstetric fistula (Table 2).

**Table 2:** Multiple covariate analysis of time to recovery from obstetric fistula patients at Gondar university teaching and referral hospital (January 2010–March, 2017)

Variables	Crud hazard ratio		Adjusted hazard ratio	
	CHR	(95% CI)	AHR	(95% CI)
Economical dependence				
Dependent	1		1	
Independent	1.92	1.62 2.29	1.05	0. 80 1.39
Delivery outcome				
Still-born	1		1	
Alive	1.35	1.13 1.60	1.16	0.89 1.52
BMI				
Normal weight	1		1	
Under weight	0.39	0.30 0.50	0.45	0.30 0.68
Over weight	0.61	0.50 0.74	0.56	0.41 0.76
Obesity	0.25	0.16 0.38	0.41	0.21 0.80
Parity				
Primiparous	1		1	
Multiparous	1.31	1.11 1.56	1.51	1.17 1.96
Delivery place				
Home	1		1	
Health service	1.48	1.25 1.75	0.95	0.73 1.25
Duration of labor	0.88	0.84 0.92	0.98	0.91 1.06
Antibiotic use				
Didn’t use	1		1	
Use	2.46	2.05 2.96	1.49	1.11 2.01
Residence				
Urban	1		1	
Rural	0.56	0.45 0.69	0.72	0.52 1.01
Antenatal care visit				
Don’t use	1		1	
Used	3.35	2.80 4.01	1.95	1.39 2.73
Physiotherapy				
No	1		1	
Yes	1.87	1.57 2.23	1.23	0.93 1.63
Duration of bladder catheterization	0.92	0.91 0.93	0.93	0.90 0.95
Length of fistula hole				
Small (< 2)	1		1	
Medium (2–3.9)	0.61	0.48 0.78	0.71	0.50 1.02
Large (4–5.9)	0.39	0.31 0.48	0.62	0.43 0.91
Extensive (>/=6)	0.23	0.16 0.322	0.42	0.23 0.78
Width of fistula hole				
Small (< 2)	1		1	
Medium (2–3.9)	0.40	0.32 0.50	0.40	0.28 0.58
Large (4–5.9)	0.31	0.24 0.39	0.50	0.33 0.75
Extensive (>/=6)	0.22	0.15 0.33	0.55	0.28 1.08
Educational status				
Illiterate	1		1	
Literate	3.97	3.26 4.84	2.23	1.62 3.06
Constant			0.01	0.005 0.021

Using antibiotic drugs is responsible for increased rate of recovery by 49% (AHR = 1.49, 95% CI = 1.11–2.01) as compared to non-use of antibiotic. The rate of recovery for patients who had ANC follow-up was higher by 95% (AHR = 1.95, 95% CI = 1.39–2.73) than patients who had no ANC follow-up history. The rate of recovery for literate patients was 2.23 times more likely (AHR = 2.23, 95% CI = 1.62–3.06) than illiterate patients. Being underweight delays time from recovery. The rate of recovery for underweight women was decreased by 55% (AHR = 0.45, 95% CI = 0.30–0.68) compared to patients who had normal weight. The rate of recovery decreased by 44% (AHR = 0.56, 95% CI = 0.41–0.76) and 59% (AHR = 0.41, 95% CI = 0.21–0.80) for overweight and obese patients respectively as compared to those with normal weight. Also the rate of recovery for multiparous patients was higher by 51% (AHR = 1.51, 95% CI = 1.17–1.96) than those who were primiparous (Table 2).

In patients who had large length of fistula, the rate of recovery was decreased by 38% (AHR = 0.62, 95% CI = 0.43–0.91) compared to those who had small length of fistula. The rate of recovery was decreased by 58% (AHR = 0.42, 95% CI = 0.23–0.78) when patients had extensive length of fistula compared to small length of fistula. Also, the rate of recovery was decreased by 60% (AHR = 0.40, 95% CI = 0.28–0.58) for patients who had medium width of fistula compared to those with small width of fistula. Furthermore, the rate of recovery was decreased by 50% (AHR = 0.50, 95% CI = 0.33–0.75) for patients who had medium width of fistula compared to those with small width of fistula. As the duration of bladder catheterization increased by a day, the rate of recovery decreased by 7% (AHR = 0.93, CI = 0.90–0.95) (Table 2). Since the variance of univariate frailty was significant for gamma distribution, using univariate frailty model to incorporate unobservable factors is acceptable. The variation of unobservable effect among individual was 0.53 (Table 3).

**Table 3:** Testing the need of univariate frailty in the model of time to recovery from obstetric fistula patients at Gondar university teaching and referral hospital (January, 2010–March, 2017)

Parameters for baseline distribution and frailty	Value	95% CI
P	3.18	2.80 3.62
Theta	0.53	0.32 0.87
LR test of theta = 0: chibar2 = 30.03 (Prob. > = ∣chibar2∣) < 0.001		

*LR* Likelihood Ratio; P = shape parameter for Weibull Distribution; Theta = variance of frailty term

## Discussion

The main goal of this study was to estimate the average recovery time and to identify its determinant factors from obstetric fistula patients. The average recovery time was 5.14 weeks. Body mass index (BMI), parity, antibiotic use, antenatal care visit, duration of bladder catheterization, length of fistula hole, width of fistula hole and educational status were significant predictors of recovery time.

The proportion of recovered patients was 88.07% (95% CI: 85.25, 90.42%). This result is in line with that of a study conducted in Addis Ababa [12]. However, this result was higher than a study conducted at Yirgalem Hamlin fistula hospital [11]. This might be due to the difference in the study period. The current study includes 7 year data but the previous study includes only 1 year data. The average recovery time was 5.14 week, which is nearly the same as studies conducted at Yirgalem Hamlin fistula hospital and Addis Ababa [11, 12].

Normal weight reduces recovery time compared to overweight, obese and underweight women. A study conducted at Yirgalem Hamlin fistula hospital [11] indicates as weight increases the recovery time from obstetric fistula also increases. The possible explanation might be that, as patient’s body weight increases, the circulatory system is stressed due to the increased amount of adipose (fat). Due to higher amount of fat accumulation the vascular system becomes overwhelmed and can’t supply the required oxygen and nutrients, as a result poor healing process and longer recovery time will happen. The other possible reason might be that underweight patients have low immune system and increased susceptibility to infection which endangers the recovery time.

Women giving birth for the first time took longer recovery time as compared to those who had more than one birth; probably in Ethiopia context mostly women giving birth for the first time are physically and mentally underdeveloped, leading to develop more complicated fistula case. When the duration of bladder catheterization was increased, the recovery time also increased. The same finding is observed on a study conducted in developing countries [15]. Moreover, this study indicates as the length and width of fistula increases, the recovery time also increases. This is in line with previous studies conducted in Addis Ababa [13, 14] and developing countries [15]. The recovery time of patients who had history of ANC follow-up was relatively shorter than patients who had not ANC follow-up, in agreement with a study conducted at Yirgalem Hamlin fistula hospital [11]. It is reasonable that ANC follow-up enables to get general counseling services and there is a tendency to come early for treatment when fistula occurs. Patients who used antibiotics have a short recovery time as compared with those who had not use antibiotic, as previously described [15]: it is reasonable that antibiotic prevents certain infections which in turn reduces fistula recovery time.

Relative to illiterate patients, literate patients had short recovery time. An institutional based study conducted in Addis Ababa [20] shows low educational level was associated with longer duration of labor and more still birth. It could be due to literate patients might have more information about the availability of treatment of obstetric fistula and then they may have early treatment. The study conducted in Addis Ababa [12] stated that circumferential fistula or involving the urethra, small residual bladder size and severe (complete vaginal scarring) were risk factors associated with repair failure. Additionally, a systematic review in developing countries [15] showed the scarring of vagina, increased degrees of urethral involvement and smaller bladder size were the risk factors for failure to close the fistula. However, the effect of these parameters could not be seen in the current study because of unavailability of documentation. Moreover, the effect of age at first marriage on the recovery time of obstetric fistula was seen at Yirgalem Hamlin fistula hospital [11]. The recovery time was relatively short for patient who got married after 20 years as compared to those who got married before 15 years. But, because of unavailability of the data in patients chart, it is not consider in the current study.

Generally, since this study was conducted using secondary data, some important predictor variables were not included in the analysis because of unavailability of the data. This is the limitation of this study.

## Conclusions

The average recovery time from obstetric fistula patients was 5.14 week. Small Length and width of fistula, patient educational status (literacy), antibiotic use, history of antenatal care visits, normal BMI, short period catheterization and multiparous were a significant determinate variables which shorten the recovery time of obstetric fistula.

## Abbreviations

AHR: Adjusted hazard ratio
AIC: Akaikie information criterion
ANC: Antenatal care
BMI: Body mass index
C: Confidence interval
CHR: Crud hazard ratio
Cm: Centimeter
IQR: Inter quartile range
Kg: Kilogram
STATA: Statistical analysis
UNFPA: United nation population fund
WAHA International: Women and health alliance international

## References

[1] Hinrichsen D. Obstetric Fistula: Ending the Silence, Easing the Suffering. INFO Reports, No. 2. Baltimore, Johns Hopkins Bloomberg School of Public Health, The INFO Project. 2004.

[2] Cattingham J, Royston E. Maternal health and safe motherhood program. Geneva: WHO; 1989.

[3] Bellows B, Bach R, Baker Z, Warren C. Barriers to obstetric fistula treatment in low-income countries: a systematic review. Trop Med Int Health. 2017;22(8):938–59. 10.1111/tmi.12893. 10.1111/tmi.12893 28510988

[4] When childbirth harms: obstetric fistula. New York: UNFPA; 2012.

[5] International WSH. Annual report. Bern: Women’s Hope International; 2015.

[6] Adler AJ, Ronsmans C, Calvert C, Filippi V (2013). Estimating the prevalence of obstetric fistula: a systematic review and meta-analysis. BMC Pregnancy Childbirth.

[7] Biadgilign S, Lakew Y, Reda AA, Deribe K (2013). A population based survey in Ethiopia using questionnaire as proxy to estimate obstetric fistula prevalence: results from demographic and health survey. Reprod Health.

[8] Central Statistical Agency (Ethiopia) (2016). Ethiopia demographic and health survey.

[9] Kimani ZM, Ogutu O, Kibe A. The prevalence and impact of obstetric fistula on women of kaptembwa-Nakuru, Kenya. Int J Appl Sci Technol 2014;4(3).

[10] International Waha (2011). Press briefing obstetric fistula in Gondar.

[11] Getachew T, Taye A, Jabessa S (2015). Survival analysis of time to recovery from obstetric fistula: a case study at yirgalem Hamlin fistula hospital, Ethiopia. J Biom Biostat.

[12] Nardos R, Browning A, Chen CCG (2009). Risk factors that predict failure after vaginal repair of obstetric vesicovaginal fistula. Obstet Gynecol.

[13] Goh JT, Browning A, Berhan B, Chang A (2008). Predicting the risk of failure of closure of obstetric fistula and residual urinary incontinence using a classification system. Int Urogynecol J.

[14] Browning A. Prevention of residual urinary incontinence following successful repair of obstetric vesico-vaginal fistula using a fibro-muscular sling. BJOG. 2004;111:357–61. 10.1111/j.1471-0528.2004.00080.x 15008773

[15] Frajzyngier V, Ruminjo J, Barone MA. Factors influencing urinary fistula repair outcomes in developing countries: a systematic review. Am J Obstet Gynecol. 2012. 10.1016/j.ajog.2012.02.006 PMC3398205 22475385

[16] Raassen TJ, Verdaasdonk EG, Vierhout ME (2008). Prospective results after first-time surgery for obstetric fistulas in east African women. Int Urogynecol J.

[17] WAHA international annual activity report. WAHA international; 2011. waha-international.org.

[18] Chow S-C, Shao J, Wang H. In: Jones B, Liu J-P, Peace KE, editors. Sample size calculations in clinical research. 2nd ed; Comparing time-to-event data. 2008.

[19] Wienke A. Frailty models in survival analysis. United States of America: Taylor and Francis Group; 2011.

[20] Muleta M, Rrasmussen S, Kiserud T (2010). Obstetric fistula in 14,928 Ethiopian women informal health care.

